# Multimodal magnetic resonance scans of patients with mild cognitive impairment

**DOI:** 10.1590/1980-5764-DN-2023-0017

**Published:** 2023-12-15

**Authors:** Yu Cui, Chenglong Liu, Ying Wang, Hongyan Xie

**Affiliations:** 1 Shandong First Medical University, The Second Affiliated Hospital, Department of Neurosurgery, Tai’an, Shandong, China.; 2 Shandong First Medical University, The Second Affiliated Hospital, Department of Radiology, Tai’an, Shandong, China.; 3 Shandong First Medical University, Department of Scientific Research, Ji’nan, Shandong, China.; 4 Shandong First Medical University, The Second Affiliated Hospital, Department of Neurology, Tai’an, Shandong, China.

**Keywords:** Magnetic Resonance Imaging, Cognitive Dysfunction, Early Diagnosis, Imageamento por Ressonância Magnética, Disfunção Cognitiva, Diagnóstico Precoce

## Abstract

The advancement of neuroimaging technology offers a pivotal reference for the early detection of mild cognitive impairment (MCI), a significant area of focus in contemporary cognitive function research. Structural MRI scans present visual and quantitative manifestations of alterations in brain tissue, whereas functional MRI scans depict the metabolic and functional state of brain tissues from diverse perspectives. As various magnetic resonance techniques possess both strengths and constraints, this review examines the methodologies and outcomes of multimodal magnetic resonance technology in MCI diagnosis, laying the groundwork for subsequent diagnostic and therapeutic interventions for MCI.

## RESUMO

O avanço da tecnologia de neuroimagem oferece uma referência fundamental para a detecção precoce do comprometimento cognitivo leve (CCL), uma área significativa de foco na pesquisa contemporânea da função cognitiva. A ressonância magnética estrutural apresenta manifestações visuais e quantitativas de alterações no tecido cerebral, enquanto a ressonância magnética funcional retrata o estado metabólico e funcional dos tecidos cerebrais sob diversas perspectivas. Como várias técnicas de ressonância magnética possuem pontos fortes e restrições, esta revisão examinou as metodologias e os resultados da tecnologia de ressonância magnética multimodal no diagnóstico de CCL, estabelecendo as bases para intervenções diagnósticas e terapêuticas subsequentes para CCL.

## INTRODUCTION

Mild cognitive impairment (MCI) is identified as an intermediary phase between healthy aging and dementia. Approximately 10–15% of individuals aged over 65 years old are affected by MCI^1^. MCI is categorized into two subtypes: amnestic mild cognitive impairment (aMCI) and non-amnestic mild cognitive impairment (naMCI). Notably, the progression rate from aMCI to Alzheimer disease (AD) surpasses that of naMCI^2^. In a study by Gemma et al., out of 3,935 MCI patients monitored over 2-3 years, 1,314 (34%) progressed to AD, 33 (0.8%) advanced to other dementia types, while 256 (6.5%) remained in the MCI stage^3^. Early classification of MCI is helpful for the preclinical detection of AD. Once MCI progresses to AD, the condition becomes irreversible, profoundly impacting the lifespan and quality of life of affected seniors. Clinical symptoms of MCI are ambiguous, and the absence of highly sensitive diagnostic tools complicates its identification. Currently, the primary method for MCI diagnosis relies on patients’ clinical presentations and neuropsychological assessments^4^.

In recent years, neuroimaging techniques such as structural magnetic resonance imaging (MRI), functional magnetic resonance imaging (fMRI), diffusion tensor imaging (DTI), diffusion weighted imaging (DWI), magnetic resonance spectroscopy imaging (MRS), and arterial spin label (ASL) have provided insights into brain activity, water molecule diffusion, and metabolite levels, among others. Furthermore, these methods assist in identifying changes related to neuronal damage, cerebral blood flow, and metabolic processes^3,5^.

### Structural magnetic resonance imaging

With the widespread use of high-field MRI, brain MRI data can be acquired quickly. High-resolution T1-weighted (T1WI) structural images allow for the quantitative analysis of patients’ gray and white matter volumes. This aids in assessing alterations in brain morphology and structure during disease progression^6^. Some studies have grouped individuals into naMCI, aMCI, and control categories and evaluated them using structural magnetic resonance imaging (sMRI)^7,8^. In the aMCI group, the volumes of the hippocampus, entorhinal cortex, and amygdala diminished, while the thickness of the cortex in the entorhinal cortex, fusiform gyrus, precuneus lobe, and cingulate isthmus decreased. In contrast, only the volume of the precuneus lobe showed a decline in the naMCI group. These findings suggest that MCI classifications can be discerned from brain structural perspectives using sMRI, aiding in predicting dementia types and associated risks. Furthermore, sMRI proves valuable in assessing the effectiveness of therapeutic medications^7^.

A longitudinal assessment of cortical atrophy, as detected by sMRI, can serve to monitor the progression of MCI^9,10^. Gemma et al. posited that although sMRI can identify early-stage atrophy in the hippocampus and medial temporal lobe, its limited sensitivity and specificity for MCI diagnosis preclude its use as a sole predictor for MCI progression to AD^3^. Future research should prioritize the combination of tests, rather than relying solely on a single modality such as sMRI, to enhance early diagnosis of MCI.

### Functional magnetic resonance imaging

fMRI, also known as blood oxygen level-dependent (BOLD) imaging, is based on the degree of influence of neuronal activity on local brain tissue oxygen consumption and cerebral blood flow so that the ratio of oxygenated hemoglobin to deoxyhemoglobin in the blood in the local area of the brain changes^11^. These changes lead to variations in MRI signals that offer insights into brain activity. A heightened blood oxygen level signifies augmented blood flow to a specific brain region, suggesting elevated activity in that area^12^. fMRI can be categorized into two types: resting-state fMRI (rs-fMRI) and task-based fMRI (tb-fMRI), based on whether a task is being performed by the subject^13^.

Given its high temporal resolution, repeatability, non-invasiveness, and absence of radioactivity, fMRI is extensively employed in neuroscience and clinical research. This modality offers novel insights and avenues for understanding and investigating neurological and psychiatric disorders^14^. Relative to sMRI and neuropsychological scales, fMRI offers a more objective assessment. To date, several methods are available for analyzing fMRI data, including regional homogeneity analysis (ReHo)^15^, amplitude of low-frequency fluctuations (ALFF)^16^, independent component analysis^17^, functional connectivity^18^, and graph theory methods^19^ among others.

### Resting-state functional magnetic resonance imaging

rs-fMRI is employed to detect spontaneous low-frequency oscillations, which arise from signals dependent on cerebral blood oxygen levels in a resting state. This technique provides insights into local brain activity and functional networks. Its advantages include simplicity, non-invasiveness, and superior spatial and temporal resolution, making it a prevalent tool for studying brain functions in neuropsychiatric disorders^20^.

Currently, rs-fMRI is primarily employed to detect functional and connectivity alterations in AD patients, MCI patients, and healthy populations. Additionally, it offers biomarkers, including neuronal dysfunction, neuronal loss, and cognitive decline in older adults, facilitating the early diagnosis and prevention of AD^21,22^. A comprehensive neuropsychological assessment was performed on MCI patients. Machine learning algorithms and cross-validation techniques were employed to evaluate the classification of MCI and healthy controls. Classification accuracy surpassed that of sMRI data, underscoring the significance of rs-fMRI in MCI identification^23^.

A meta-analysis examining rs-fMRI data from aMCI and AD patients utilized various methods, including regional uniformity, low-frequency fluctuation amplitude, amplitude fraction, and whole-brain connectivity. Findings revealed reduced functional features in the left hippocampal gyrus of AD patients. Certain detection parameters, such as local consistency, low-frequency amplitude value, and whole-brain network connection, exhibited minor changes. Both aMCI and AD patients demonstrated a consistent decline in these parameter values^24^.

### Functional connection network

Functional connectivity denotes the synchronized or correlated brain activity across two or more functional brain regions over time, laying the theoretical groundwork for exploring complex brain networks through graph theory^25^. For MCI, assessing cognitive deficits using fMRI-derived brain functional connectivity offers a dependable means to elucidate the disease’s fundamental pathophysiological mechanisms and estimate its progression stage^26^. In a three-year longitudinal study, rs-fMRI was used to assess the functional connectivity of relevant brain areas in 23 MCI patients. Of these, 7 patients progressed to AD, while 14 remained cognitively stable^27^.

Impaired functional connectivity in the temporal lobe, particularly the hippocampus and parahippocampal gyrus, as well as the loss of parahippocampal white matter volume in MCI patients, are linked to memory deficits observed in both MCI and AD patients. These factors are viewed as predictors for MCI and AD^28^. Numerous rs-fMRI studies employing graph theory techniques have identified small-world network characteristics in the functional connectivity networks of MCI patients’ brains^29^. Numerous studies indicate that there is a disruption in the whole-brain topological organization of the functional connectome in MCI patients. This includes disruptions in functional activity across expansive networks or interconnected brain regions. Compared to a normal network, the small-world network property of an MCI patient’s brain functional network has altered. This suggests that the small-world network property might offer a valuable foundation for the early diagnosis, differential diagnosis, and efficacy evaluation of MCI and AD^30,31^.

### The default-mode network

Currently, the default-mode network (DMN) is the primary network evaluated using rs-fMRI for studying cognitive functions. It is associated with episodic memory, executive function, and various cognitive and emotional changes^27^. DMN is subdivided into anterior DMN — which includes the medial prefrontal cortex, dorsal prefrontal cortex, anterior cingulate gyrus, and lateral temporal lobe — and posterior DMN — encompassing the ventral prefrontal cortex, posterior cingulate gyrus, parietal lobule, gyrus, hippocampus, and medial temporal lobe^32^. Functional connectivity alterations in DMNs were observed among healthy aged individuals, and MCI and AD patients^33^. Using independent component analysis, Damoiseaux et al. observed a decrease in DMN posterior connectivity and an increase in ventral and anterior connectivity among MCI patients^34^. Gardini et al. identified heightened DMN connectivity in MCI patients between the medial prefrontal lobe and several regions, including the posterior cingulate gyrus, parahippocampus, and anterior hippocampus. They hypothesized that this could be attributed to maladaptive mechanisms^35^.

Some studies suggested that DMN functional connectivity decreases with age, while Dennis’s study suggested that compensatory mechanisms during aging may cause DMN connectivity to increase with age; the authors speculated that this may be due to compensatory mechanisms during aging^36^. As cognitive impairment intensified, DMN connectivity in the posterior cingulate/anterior precuneus region diminished^37^. In healthy individuals, DMN and the central executive network consistently exhibit opposing activities, both at rest and during task performance^38^. DMN is believed to be more active during internally directed cognitive activities, such as self-monitoring and social functions, whereas the central-executive network is predominantly activated during externally directed higher-order cognitive functions like attention, working memory, and decision-making^39^.

The dynamic control of the switch between DMN and central-executive networks is an evolving area of research. A recently suggested triple network model incorporates fMRI to compare these two antagonistic networks and introduces a third component, the salience network^40^. This model aims to elucidate the connectivity patterns observed in cognitively intact brains and the alterations evident in cognitive impairments^41^. In healthy individuals, the salience network has been identified as pivotal in dynamically modulating the antagonistic activity between DMN and central-executive networks^42^. Yet, it remains uncertain whether this dynamic modulation persists in normal aging or if it changes in the presence of MCI.

Ganesh et al. utilized rs-fMRI to explore the interplay between MCI and the tripartite network structure observed in the standard population. Their findings indicate that in MCI patients, alongside changes in interaction with the central executive network, there is also dysfunction in the salience network. Intriguingly, the severity of salience network dysfunction was found to correlate with the degree of overall cognitive decline. Hence, the salience network emerges as a potential neuroimaging marker for cognitive impairment^43^.

Some studies have applied fMRI to compare MCI disease and AD, tracking patients for a span of five years. Results indicate that hippocampal activation levels can be utilized as markers of cognitive deterioration. Specifically, elevated activity in this area is associated with significant cognitive decline and a heightened likelihood of MCI patients progressing to dementia^44^. Yetkin et al. examined visual memory functions across three groups: MCI patients, AD patients, and healthy controls. Their findings revealed that both MCI and AD patients displayed significantly heightened activity in specific functional regions when contrasted with healthy controls. Primarily, this increased activity encompassed the right frontal superior gyrus, bilateral middle temporal gyrus, middle frontal gyrus, and the anterior segment of the bilateral cingulate gyrus. These observed activity patterns in unique functional zones present crucial insights into the progression of MCI and may serve as a foundational framework for disease diagnosis^45^.

### Task-based functional magnetic resonance imaging

In tb-fMRI, time series data are compared against a hypothesized model of neural function based upon the cognitive task being performed. Through the use of statistical inference, the hypothesis can be accepted or rejected for every voxel. In this way, a map of those brain regions that respond to the task is constructed^46^. Jacobs et al. found that the activation of dorsal and ventral pathways in patients with aMCI increased, activation of the medial and lateral parietal lobes decreased, and activation of the parietal and temporal lobes increased when performing tasks such as object recognition^47^. Dorsal pathway dysfunction is considered to be the anatomical basis of visual space dysfunction in patients with MCI and AD.

Brain regions as adjacent lesions associated with visual space processing are mainly concentrated in the frontal parietal lobe, including two independent systems of the ventral pathway and dorsal pathway: ventral pathways are composed of lateral temporal lobes and temporal occipital lobes, which are mainly responsible for the recognition of object shapes; dorsal pathways are composed of three sub pathways — apical lobe projections to the medial temporal lobe, prefrontal lobe, and anterior motor region, which are mainly responsible for sensing and identifying objects seen by the eyes and the storage of visual spatial memory in the medial temporal lobe and hippocampus^48,49^.

### Diffusion weighted imaging

DWI primarily assesses the microscopic movement of water molecules within living tissues. This diffusion rate, especially in instances of slowed water molecule movement within the tissue, is represented by the apparent diffusion coefficient (ADC). A reduced ADC value results in a darker image appearance.

Kumar et al. carried out various examinations, including simple mental status tests, DWI, DTI, and more, on both MCI patients and control groups^50^. Their findings revealed significant changes in ADC values in the right temporal lobe, hippocampus, callosum, and other regions of MCI patients. In a separate study by Bergamino et al., DWI scans and cognitive evaluations were performed on 12 MCI patients, 13 AD patients, and 24 healthy individuals^51^. The results demonstrated significant alterations in ADC values of the amygdala and hippocampus in MCI patients compared to healthy controls. These findings suggest that DWI indicators have the potential to serve as biomarkers for MCI.

A prior study discovered that ADC values in the cerebral cortex and hippocampus of MCI patients were significantly elevated compared to healthy volunteers. Furthermore, these ADC values directly correlated with the severity of cognitive impairment^52^. In a separate study, Kantarci et al. monitored 21 MCI patients and observed that, despite the absence of hippocampal structural atrophy, there were changes in ADC values^53^. Higher ADC values in the hippocampus were associated with an increased likelihood of the patient progressing to AD. This suggests that the ADC value in the hippocampus could be a predictive measure for MCI transitioning to AD, offering potentially more valuable insights than structural MRI data alone.

### Diffusion tensor imaging

DTI assesses the orientation and integrity of white matter tracts by measuring the diffusion rate and direction of water molecules. This reveals the condition of the white matter fiber bundles and their anatomical associations with adjacent lesions^54^. DTI primarily utilizes parameters such as fractional anisotropy (FA), mean diffusivity (MD), and ADC. In fact, FA reflects the preferential direction in which water molecules can diffuse. If there is no preferred direction, water molecules can equally diffuse in all directions, so that FA is zero, *i.e*. there is no preferred direction. In regions where FA is close to 1, it means that there is a preferential direction for the water molecules. Anatomically, for white matter fibers, water molecules can diffuse only in the direction of the fibers^55^. FA offers insights into the density of myelin and the structural wholeness of fibrous tracts. Its value lies between 0 and 1, where a greater FA value signals superior nerve conduction. Conversely, a diminished FA value signifies notable white matter deterioration. MD predominantly measures the velocity and extent of water molecule diffusion in tissues. Elevated MD values suggest enhanced water molecule diffusion capability and a heightened degradation of fibers integrity^56^.

In a comprehensive study, 33 patients with aMCI, 15 with AD, and 20 healthy controls underwent evaluations using structural MRI, DTI, and MRS. Results indicated that DTI emerged as the most sensitive diagnostic tool for identifying MCI, boasting a sensitivity of 90.9% and a specificity of 50%. Additionally, when differentiating between MCI and AD, DTI achieved the highest specificity, reaching 87.9%^57^.

In comparison to cognitively normal individuals, MCI patients exhibited a decrease in the FA value in the medial temporal lobe and an elevated MD value^58^. These findings suggest an impairment in the integrity of white matter fibers. Decreased FA values in brain regions, including the corpus callosum, corona radiata, and cingulate gyrus, have been linked to cognitive impairments^59^. FA values in the parietal and temporal lobes, as well as MD values in the corpus callosum, are correlated with the global cognitive abilities and episodic memory in MCI patients^60^. This suggests that DTI metrics in these brain regions can serve as reliable markers for assessing the cognitive status of MCI patients.

Shim et al. discovered that changes in the white matter microstructure occurred before hippocampal atrophy in MCI patients^61^. By comparing the volume of the hippocampus with white matter integrity, they suggested that assessment of white matter health by DTI could serve as an imaging marker for cognitive decline and MCI diagnosis. This could pave the way for its use as a potent clinical tool for early AD diagnosis and monitoring disease progression.

In a longitudinal study of 132 MCI patients, structural MRI, DTI, and positron emission tomography (PET) scans were utilized^62^. Findings indicated that fractional anisotropy of the genu of the corpus callosum (FA-Genu) could serve as a predictor of cognitive decline severity in MCI patients. Notably, DTI, specifically FA-Genu, offered invaluable complementary insights to established AD biomarkers and underscored their potential in anticipating cognitive deterioration in MCI. In a 2.5-year longitudinal study involving 23 MCI patients, Mielke et al. discovered a correlation between FA and MD values of the vault in 6 MCI patients who later developed AD and the hippocampal volume^63^. Furthermore, the DTI value of the vault appeared to be a predictive marker for memory deterioration in MCI patients.

Liu et al. employed spatial statistical analysis in tandem with fiber bundle tracking to conduct DTI on patients with aMCI^64^. Their findings revealed decreased FA values across several brain regions and heightened MD values in the frontal, parietal, and temporal lobes. Sali Dimitra et al. studied 19 aMCI patients with impairments across multiple cognitive domains^65^. After utilizing a comprehensive neuropsychological assessment and analyzing DTI data, they discerned that FA values of the callosum, posterior cingulate, anterior cingulate, and superior longitudinal bundles in these patients were markedly lower compared to a healthy control group. They posited that the integrity of the white matter fiber bundles was compromised in aMCI patients, leading to cognitive deficits.

Extant literature on aMCI and AD patients reveals that when DTI was employed to assess cerebral white matter fiber bundles, a significant decrease in the FA value of the cingulate girdle was observed in aMCI patients^2^. In contrast, AD patients demonstrated decreased FA values in several other brain regions, including the prefrontal lobe, temporal lobe, and hippocampus^66^.

### Diffusion kurtosis imaging

Diffusion kurtosis imaging (DKI) is an advanced technique derived from DTI that elucidates the non-Gaussian diffusion of water molecules within tissues, allowing for a more detailed representation of tissue microstructure than its predecessor^67^. The primary parameters of DKI encompass mean kurtosis (MK), MD, radial kurtosis, and kurtosis anisotropy^68^. Notably, MK reveals the non-Gaussian diffusion characteristics in both white and gray matters, thereby aiding in a comprehensive depiction of microstructural variations in white matter tracts and deep gray matter regions^69^.

In the context of diagnosing aMCI and AD, it is posited that alterations in DKI parameters, especially those of the bilateral hippocampus, are more indicative than mere hippocampal atrophy, positioning DKI as a superior tool in comparison to DTI^70^. Zhang et al. emphasized that in the prodromal phase of dementia, the predominant changes were in hippocampal microstructures^71^. The most salient discriminators turned out to be microstructural measurements: left hippocampal MK for subjective cognitive decline and right hippocampal MD for MCI. Furthermore, DKI distinctly highlights alterations in tissue microstructures, particularly within deep gray matter nuclei. Intriguingly, changes in DKI parameters manifest prior to any discernible shifts in brain morphology among MCI patients.

### Magnetic resonance spectroscopy imaging

MRS is a non-invasive method that detects energy metabolism and biochemical alterations in living tissues. This technique provides valuable metabolic information about tissues, making it instrumental for the early diagnosis and differentiation of MCI.^72^ The primary metabolites employed for diagnostic purposes encompass N-acetyl aspartate (NAA), choline-containing compounds (Cho), myoinositol (MI), and creatine (Cr). NAA predominantly resides in the mitochondria of neurons and serves as an indicator of neuronal and axonal density^73^. A diminished NAA level in gray matter suggests neuronal loss and metabolic changes, whereas a decline in white matter implies axonal damage. Cho plays a pivotal role in the synthesis of cell membranes and myelin sheaths; a decreased level signifies sphingomyelin breakdown and cell membrane disintegration. MI acts as a glial cell marker, and an increase in its concentration can indicate glial hyperplasia. Conversely, Cr content remains relatively consistent, often used as a reference to monitor fluctuations in other metabolite levels^74^.

A study demonstrated that the diminished NAA concentration level in the hippocampus of MCI patients falls between that observed in AD patients and healthy aged individuals. Furthermore, this decrease in NAA was inversely proportional to the severity of their memory impairment, positioning MCI as an intermediate state between normal aging and AD^75^. Kantarci et al. tracked 1,156 cognitively normal individuals for an average duration of 2.8 years, during which 214 participants progressed to MCI or dementia. Their findings underscored that both hippocampal volume reduction and variations in the NAA/MI ratio served as independent predictors of MCI^76^.

Mitolo et al. embarked on a 2-year follow-up study involving 38 MCI patients, 23 AD patients, and 18 healthy controls. They deduced that jointly utilizing the NAA/MI ratio and the volume of the parahippocampal gyrus could foretell the rate of AD conversion, boasting an impressive sensitivity of 84.6% and a specificity of 91.7%^77^. Another research initiative that combined MRI and MRS to anticipate the progression from MCI to AD observed that, out of 214 healthy aging adults, a significant fraction progressed to either MCI or dementia over a span of 2.8 years^76^. This progression was determined by conducting single-voxel proton MRS of the posterior cingulate gyrus and using MRI to evaluate both hippocampal volume and white matter hyperintensity volumes.

A study employed MRS to evaluate 13 patients diagnosed with aMCI according to the Mayo Clinical Medical Center criteria^78^. Upon analyzing the NAA/MI, NAA/Cr, Cho/Cr, and MI/Cr ratios in both cingulate areas, it was discerned that the MI/Cr ratios in the anterior cingulate gyrus regions differed notably between the two sides in aMCI patients. This asymmetry was subsequently deemed a crucial biomarker for aMCI.

Zhao et al. assessed 69 MCI patients alongside 67 healthy controls. They calculated the NAA/Cr and Cho/Cr ratios of the bilateral hippocampus and posterior cingulate gyrus and examined the relationships between these ratios and Mini-Mental State Examination (MMSE) scores^79^. Their findings indicate that MCI could manifest when the NAA/Cr ratio is less than 1.19 in either the left or right hippocampus.

Kantarci et al. selected a diverse group comprising 21 MCI patients, 21 AD patients, and 63 healthy controls. Utilizing MRS, they conducted a metabolic analysis of the upper temporal gyrus, posterior cingulate gyrus, and medial parietal lobe^53^. Their analysis revealed a lower NAA/Cr ratio in the left temporal upper gyrus and posterior cingulate gyrus of AD patients compared to the MCI and healthy control groups. Furthermore, both the MCI and AD groups exhibited a higher MI/Cr ratio in the posterior cingulate gyrus than healthy controls. Additionally, the AD group displayed a heightened Cho/Cr ratio in the posterior cingulate gyrus compared to both MCI patients and healthy controls. An elevated MI/Cr ratio suggests glial hyperplasia and a progression from MCI to AD, while a decline in the NAA/Cr ratio coupled with a rise in the Cho/Cr ratio indicates an advanced stage of the disease. It was posited that the cingulate cortex NAA/Cr ratio post-MRS observation might be the most discerning method to differentiate between AD and MCI.

Another research endeavor followed a cohort of sex- and age-matched MCI patients for 18 months^80^. It was observed that the NAA/Cr ratio in the posterior cingulate gyrus of patients transitioning to AD was lower compared to those evolving into Lewy body dementia (LBD), highlighting the potential of MRS in predicting the progression direction of MCI.

### Arterial spin label

The cerebral blood flow (CBF) indicates the volume of blood that circulates through a specific cross-sectional area of cerebral vessels within a given time frame. This parameter is known to decline with age. By utilizing magnetically labeled arterial blood as an intrinsic contrast agent, it is feasible to directly and quantitatively measure CBF, providing insights into the capillary dynamics and, in turn, shedding light on the perfusion and functionality of brain tissue^81^. As a barometer, CBF can serve as a potential predictor of cognitive performance in aged people.

Recently, ASL, an innovative magnetic resonance perfusion imaging technique, has come to the fore. This modality boasts multiple benefits, such as the absence of any need for contrast medium injections, freedom from radiation, reproducibility, superior spatial resolution, brief data collection duration, and no disturbance by the blood-brain barrier. These attributes make ASL an appealing, cost-effective, and safer counterpart to positron emission tomography for clinical research applications^82^. Given its capabilities, ASL has been extensively employed to detect and monitor the initial vascular perfusion shifts in MCI patients.

ASL perfusion maps revealed varying degrees of hypoperfusion in distinct regions of the brains of MCI patients. Johnson et al. initially utilized ASL to identify a decrease in CBF within the right inferior parietal lobe of these patients^83^. Subsequent research has indicated a correlation between the extent of CBF decline and disease severity^84^. Furthermore, Camargo et al. observed a notable CBF reduction in areas such as the hippocampus, middle temporal lobe, ventral striatum, prefrontal cortex, and cerebellar regions in MCI patients^85^. Conversely, an elevation in CBF was detected in the left hippocampus, right amygdala, and basal ganglia regions, encompassing the caudate nucleus, shell nucleus, and globus pallidus^86^.

Soman et al. observed a pronounced decrease in CBF within the posterior cingulate gyrus, glossal gyrus, and hippocampus of MCI patients^87^. There was a significant correlation between overall cognition and CBF alterations in the anterior wedge and temporal neocortex. Moreover, the severity of memory decline exhibited a direct positive relationship with the extent of CBF reduction in the medial temporal lobe. In contrast, Thomas et al. identified an initial increase in CBF in the hippocampus, inferior parietal lobe, and temporal lobe of MCI patients^88^. As the condition advanced, CBF in the temporal lobe diminished. They posited an inverted U-shaped trajectory in the CBF signal within pertinent brain regions of MCI patients, indicative of early neurovascular dysfunction and a heightened CBF to offset cognitive decline. Eventually, these patients transitioned into a decompensatory phase. Despite the disparities in study results, often attributed to methodological variances and participant heterogeneity, the pathological evolution of MCI remains complex, with potential compensatory interactions between cells and blood vessels during early stages.

While hippocampal atrophy stands as a recognized indicator of AD progression, MCI patients often display no atrophy in regions like the hippocampus and medial temporal lobe. However, they do exhibit abnormal cerebrovascular functions. A longitudinal study leveraging ASL technology to anticipate cognitive shifts in the aged deduced that among all brain regions, cerebral blood flow alterations in the frontal lobe were most predictive of cognitive changes. Moreover, aberrant network patterns in the medial frontal lobe and anterior cingulate cortex emerged as crucial predictors of these cognitive variations^82^. Li et al. employed voxel analysis, revealing that the hypoperfusion in areas like the frontal lobe, medial frontal cortex, and anterior cingulate cortex were most indicative of individual cognitive predictions, closely linking to the pathophysiology of MCI^89^. Such findings underscore their potential as robust markers for assessing initial stages of cognitive decline. Given the prognostic capability of CBF regarding cognitive alterations, it holds profound implications for forecasting cognitive functions in the aged and facilitating early clinical detection and diagnosis of MCI.

In conclusion, early diagnosis and timely intervention in MCI patients may decrease the likelihood of progression to dementia. Currently, there is no established neuroimaging reference indicator for MCI identification. Techniques like structural MRI, fMRI, DTI, DWI, MRS, and ASL offer non-invasive, accurate, and high-resolution imaging with repeatability. However, individual MRI methods have limitations in isolated MCI diagnostics and differential diagnosis. Studies on cognitive function progression using rs-fMRI present both consistent and divergent results. These inconsistencies might arise from compensatory bodily mechanisms, variations in seed point selection, methodological differences, or clinical heterogeneity. While rs-fMRI is sensitive to early MCI and AD diagnosis, its predictive value requires further validation. DWI application is limited by its need for high magnetic field strength and artifacts near skull-based brain lesions. Moreover, diseases like brain tumors manifest diversely in DWI scans due to varied internal components. MRS, though adept at studying brain molecular processes without ionizing radiation, suffers from low sensitivity. ASL quantifies CBF, minimizing individual vascular differences, but its low signal-to-noise ratio compromises image quality, making it less ideal for routine clinical use^90^.

By employing multimodal MRI technology, researchers can harness complementary benefits, enabling a holistic examination of MCI from morphological, functional, and metabolic viewpoints. Such an approach provides a comprehensive diagnostic system for MCI, enriches the current understanding of its onset and progression, guides clinicians in timely MCI patient identification and treatment, and aids in preventing progression to AD. Future studies should expand sample sizes in multimodal imaging investigations, delve deeper into MCI pathophysiology from varied perspectives, and develop an MCI imaging differential diagnosis system characterized by high sensitivity and specificity.

We have summarized the advantages and disadvantages of each method in Table 1.

**Table 1:** Contrast of advantages and disadvantages.

Imaging modality	Advantages	Disadvantages
sMRI	Using sMRI, MCI can be categorized based on brain structure, such as early-stage hippocampal and medial temporal lobe atrophy. This classification aids in predicting the specific type and risk of dementia. Additionally, sMRI can assist in evaluating the effectiveness of therapeutic medications.	Limitations of sMRI include its low sensitivity and specificity in diagnosing MCI. As a result, it cannot independently and precisely predict the progression from MCI to AD.
fMRI	This technique is non-invasive, straightforward, and provides enhanced spatial and temporal resolution. The activation patterns of different functional brain areas can further assist in predicting the progression of MCI, offering a pivotal basis for diagnosing the disease.	Given the consistency and variations in study findings, further research is imperative to enhance the value of early diagnosis.
DWI	DWI stands as the sole non-invasive magnetic resonance technique capable of detecting the motion of water molecules within living tissues. DTI is utilized to evaluate the integrity of white matter fibers throughout the brain. DKI vividly illustrates changes in tissue microstructure, particularly in deep gray matter nuclei. They are valuable tools for screening MCI and forecasting its progression to dementia.	DWI’s high sensitivity to movement, combined with its lengthy examination and scanning process, can result in skewed outcomes, limiting its clinical utility.
MRS	Metabolic alterations often manifest before structural ones. As a result, MRS has the capacity to identify metabolic anomalies undetectable by conventional MRI, underscoring its significance in the early diagnosis of MCI.	Although MRS allows for noninvasive examination of molecular activities in the brain without subjecting individuals to ionizing radiation, its efficacy is curtailed by its limited sensitivity.
ASL	ASL boasts several key advantages, including the absence of contrast media injections, no radiation exposure, repeatability, brief acquisition time, and uninfluenced performance by the blood-brain barrier. Given these merits, ASL emerges as a potentially cost-effective, safe, and practical alternative to positron emission tomography in clinical research. Its utility is especially evident in detecting and monitoring early vascular perfusion changes associated with MCI.	ASL minimizes individual variations in vascular characteristics. However, these variations can result in changes in signal intensity that clinicians might misinterpret as disease-induced abnormalities. One notable limitation of ASL in clinical application is its low signal-to-noise ratio, resulting in diminished image quality.

Abbreviations: sMRI, structural magnetic resonance imaging; MCI, mild cognitive impairment; AD, Alzheimer’s disease; fMRI, functional magnetic resonance imaging; DWI, Diffusion-weighted imaging; DTI, Diffusion Tensor Imaging; DKI, Diffusion kurtosis imaging; MRS, Magnetic Resonance Spectroscopy; ASL, Arterial Spin Labeling.
